# Aesthetic Evaluation of Breast Reconstruction with Autologous Fat Transfer vs. Implants

**DOI:** 10.1007/s00266-022-03076-2

**Published:** 2022-09-13

**Authors:** Jamilla L. M. Wederfoort, Alieske Kleeven, Juliette E. Hommes, Sander M. J. Van Kuijk, René R. W. J. van der Hulst, Andrzej Piatkowski, M.D for The Breast trial investigators, Andrzej Piatkowski, Jamilla L. M. Wederfoort, Juliette E. Hommes, Sander J. Schop, Todor K. Krastev, Sander M. J. van Kuijk, René R. W. J. van der Hulst, Daniëlle Derks, Mikko Larsen, Hinne Rakhorst, Ute Schmidbauer, Jan Maerten Smit, Liang T. Tan, Kim M. E. Wehrens, Thijs de Wit

**Affiliations:** 1 Department of Plastic-, Reconstructive-, and Hand Surgery, Maastricht University Medical Center+, P.O. Box 5800, 6202 AZ Maastricht, The Netherlands; 2 NUTRIM School of Nutrition and Translational Research in Metabolism, Maastricht University, Maastricht, The Netherlands; 3 Faculty of Health, Medicine, and Life Sciences, Maastricht University, Maastricht, The Netherlands; 4 Department of Clinical Epidemiology and Medical Technology Assessment (KEMTA), Maastricht University Medical Center, Maastricht, The Netherlands; 5 Department of Plastic Surgery, VieCuri Medical Center, Venlo, The Netherlands

**Keywords:** Autologous fat grafting, Breast reconstruction, Lipectomy, Implants, Cosmetic evaluation, Aesthetic outcome

## Abstract

**Background:**

Autologous fat transfer (AFT) seems to be a new minimal invasive method for total breast reconstruction, yet how patients, surgeons, and laymen evaluate cosmesis is lacking. The aim of this study was to evaluate the aesthetic outcome of AFT (intervention group) for total breast reconstruction post-mastectomy, as compared to implant-based reconstruction (IBR) (control group).

**Methods:**

A random and blinded 3D photographic aesthetic outcome study was performed on a selection of 50 patients, scored by three panels: plastic surgeons, breast cancer patients, and laymen. Secondary outcomes included agreement within groups and possible patient characteristics influencing scoring.

**Results:**

Breast cancer patients and plastic surgeons did not differ in the aesthetic scores between the treatment groups. In contrast, the laymen group scored AFT patients lower than IBR patients (− 1.04, *p* < 0.001). Remarkably, mean given scores were low for all groups and overall agreement within groups was poor (ICC < 0.50). Higher scores were given when subjects underwent a bilateral reconstruction and if a mamilla was present.

**Conclusion:**

Evaluation of aesthetic outcomes varies greatly. Hence, aesthetic outcome remains a very personal measure and this emphasizes the importance of thorough patient counseling including information on achievable aesthetic results before starting a reconstructive procedure.

**Level of Evidence III:**

This journal requires that authors assign a level of evidence to each article. For a full description of these Evidence-Based Medicine ratings, please refer to the Table of Contents or the online Instructions to Authors www.springer.com/00266 .

## Introduction

Breast cancer is one of the most commonly diagnosed malignancy in the world, with more than two million diagnoses in 2020 [1, 2]. Current surgical approaches consist of breast-conserving surgery (BCS) with the possibility for radiotherapy and chemotherapy, or a mastectomy [3, 4]. The full treatment is multidisciplinary and personalized. The literature shows that there is a trend of preference for a mastectomy, even when breast-conserving therapy is an option for these patients [3, 5, 6]. Yet a mastectomy can be a huge disadvantage for women, as they can suffer from several psychological and physical problems due to the removal of their breast(s). Feelings of pain, an altered body image, diminished self-worth, loss of sense of femininity and sexuality, as well as anxiety have been described postoperatively [6–8].

Fortunately, breast reconstruction has shown to have multiple psychological benefits and therefore improve the patients’ quality of life (QoL). It is therefore no surprise that there is an increasing trend in patients opting for breast reconstruction post-mastectomy. Currently, about 42% of all mastectomy patients opt for breast reconstruction post-mastectomy [6, 8–10]. As every type of reconstruction has its advantages and disadvantages, more types of reconstruction methods are being researched to further refine personalized treatment.

Today, women can opt to have their breasts reconstructed with either an implant or with autologous tissue. Autologous fat grafting (AFT) is an autologous option for breast reconstruction and in the past few decades the use of AFT for total breast reconstruction has gained much attention. Nevertheless, there is still much to be explored in the field of AFT in comparison with implant-based reconstruction (IBR) and other free flap reconstructions (FFR) [4, 8, 11, 12].

Besides safety issues, QoL, and satisfaction with outcome, the aesthetic appearance of the breast is an important, if not the most important outcome measure for patients and health care providers. As AFT for total breast reconstruction is relatively new, there is no information regarding the cosmesis of AFT versus other types of breast reconstruction methods. This study aimed to obtain this missing information on AFT and evaluate the aesthetic outcome of AFT for total breast reconstruction post-mastectomy compared to IBR.

## Methods

An informed consent was obtained from all participants and evaluators. The study was ethically approved by the Medical Ethical Committee of the Academic Hospital Maastricht/ University of Maastricht (METC14-2059) and is registered at ClinicalTrials.gov (NCT02339779). The STROBE guidelines were adhered to for writing this article (Appendix A).

### Study Design

We conducted an aesthetic outcome evaluation study, in which we used anonymous images of patients who participated in the BREAST trial [13, 14]. Patients who had finished their reconstructive procedures at least 12 months or longer ago were considered eligible. From these patients, a random selection of 50 patients were chosen (25 patients from each reconstruction group). For the evaluation, post-mastectomy and 12 months postoperative 3D images were obtained. These images were then presented in a random order in a PowerPoint presentation, without treatment label. Three panels consisting of ten plastic surgeons, ten breast cancer patients, and ten age-matched male and female laymen were then requested to score the presented patient images using a visual analog score (VAS).

### Setting

All 3D images of BREAST-trial participants were gathered from seven participating centers across the Netherlands. Selection of images for this study was performed on April 30th, 2021. Thereafter, scoring of images took place between the 1st of May 2021 and the 1st of July 2021 at Maastricht University Medical Center+ (MUMC+).

### Subjects

The BREAST trial is an ongoing multicenter randomized controlled trial comparing AFT (intervention group) with IBR (control group), running from November 2015 to approximately October 2025, with the last reconstruction surgery performed in October 2020. The primary outcome for this study is the QoL, measured 12 months after the final reconstruction surgery. Moreover, the efficacy and safety of these reconstruction methods are studied [13].

To be eligible for participation in this aesthetic outcome study of the BREAST trial, patients had to meet the inclusion and exclusion criteria of the BREAST trial and be at least 12 months after their final reconstruction surgery (Table 1). Both the post-mastectomy 3D images and 12 months postoperative 3D images had to be available for presentation. Baseline characteristics including age, BMI, laterality of reconstruction, presence of mamilla, and amount of surgeries performed were gathered from the electronic patient record.

**Table 1.** Inclusion and exclusion criteria for the BREAST trial

Inclusion criteria
– Female gender
– Age 18 years or older
– Has been a candidate in the history, or is a candidate for a mastectomy in the near future
– Patients undergoing a preventive mastectomy
– It is the patient's choice to undergo breast reconstruction
– Patient wants to participate in this study
– Patient is able to wear the BRAVA^®^ device

Exclusion criteria
– Active smoker or history of smoking 4 weeks before surgery
– Current drug abuse
– History of allergy to lidocaine
– History of silicone allergy
– 4 weeks or less after chemotherapy
– History of radiation therapy in the breast area
– Oncological treatment includes radiotherapy after mastectomy
– Kidney disease
– Steroid-dependent asthma (daily or weekly) or other diseases
– Immune-suppressed or immune-compromised disease
– Uncontrolled diabetes
– BMI > 30
– Large breast size (i.e., larger than cup C), unless the patient chooses to reduce the contralateral side towards cup C
– Extra-capsular silicone leaking from the encapsulated implant as a result of previous breast reconstruction
– The plastic surgeon treating the patient has serious doubts about the patient's compliance

### Aesthetic Evaluation

#### Panels

Three evaluating groups were assigned to score images presented to them in the MUMC+, comprising a total of 30 evaluators. One group consisted of ten plastic surgeons experienced in IBR and FFR or pedicle flap reconstruction for breast reconstruction, yet not involved with the BREAST trial, meaning they had no prior experience with AFT for total breast reconstruction in post-mastectomy patients. These reconstructive surgeons in the region were invited by mail to participate in this study. The second group comprised ten breast cancer patients, regardless of what treatment they received in the past. These breast cancer patients were invited by the Dutch Breast Cancer Association (BVN). One exclusion criterion was if they participated in the BREAST trial. The last group consisted of ten laymen. These were randomly chosen colleagues of other departments and neighbors of J.W and A.K who had no prior experience with breast cancer or breast reconstruction.

#### Evaluation of the 3D Images

**Fig. 1.**
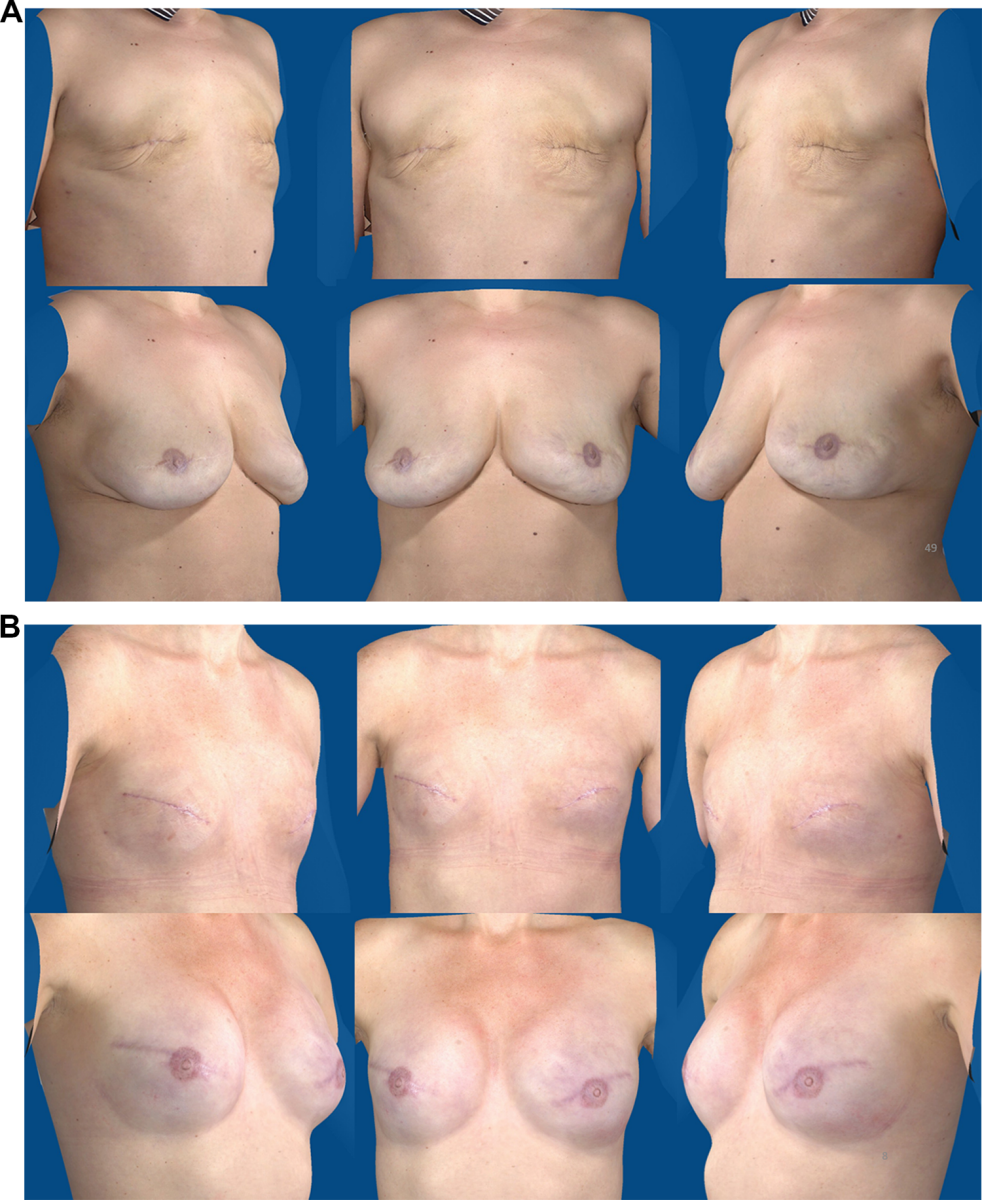
**A**, **B** Examples of presented images. The top three images on a slide showed the patient before her breast reconstruction, the lower three images showed the patient 12 months after her final reconstruction. **A** Female AFT patient, 36 years. **B** Female IBR patient*, 34 years. *For implant-based reconstruction patients, no images were available directly after mastectomy since a tissue expander (TE) was placed during the same surgery. For these patients, reference images included a tissue expander

Images of 50 patients were displayed in a PowerPoint presentation. Examples of presentation are shown in Fig. 1A, B. The R program (version 4.0.4) was used to obtain a random selection of patients to be evaluated. The top half of the slide comprised three post-mastectomy photographs display a frontal view and two-third lateral view of the patient. The lower half of the slide consisted of three 3D images taken at 12 months, post-final breast reconstruction surgery, using the same views. In this way, one slide presented both the before and after breast reconstruction state.

Ten different versions of the presentation were made, each containing a different patient order using the PowerPoint program. The evaluators needed approximately 20 minutes to score a total of 50 patients.

One of two independent researchers (J.W and A.K) displayed a version of the presentation to each evaluator. The evaluator was then asked to rate aesthetic outcome of these 50 patients, using a visual analogue score (VAS) ranging from one to ten. A score of one indicated breast reconstruction could not have been performed worse, and a score of ten indicated breast reconstruction could not have been performed better. All scoring numbers between one and ten were left unlabeled. The scoring form is shown in Appendix B. The panels were blinded for patient information and treatment type.

#### Statistical Methods

Statistical analyses were performed using SPSS IBM version 25. To present patient and evaluator characteristics, categorical variables are shown by frequencies and percentages, whereas continuous variables (age, BMI, follow-up, aesthetic scores) are presented by mean and standard deviation (SD). To conduct statistical significance of the differences between the groups, aesthetic scores were examined using independent samples *t*-tests.

The intraclass correlation coefficient (ICC) was used to calculate the interrater agreement within the groups [15]. The ICC is presented by mean and the 95 % confidence interval (CI). Because both subject and evaluator effects could be random, a random two-way random-effects model was utilized to track consistency within the panels. To assess whether a particular subject characteristic had an influence on aesthetic scoring, linear regression analysis was performed. All predictors are presented with adjusted *R*^2^ values and *p*-values.

#### Bias

In order to avoid selection bias by researchers, a random selection of 50 subjects was made by using a computer program. Subsequently, ten different versions of the PowerPoint presentation were created to prevent order bias. A selection of 50 subjects to be evaluated was deemed appropriate for sample size while avoiding evaluator fatigue. Images were not labeled for type of reconstruction. However, some reconstruction features could be identified, especially by plastic surgeons or breast cancer patients.

### Results

#### Subjects

A total of 93 patients (44 AFT, 49 IBR) met the inclusion and exclusion criteria for this study. Of these patients, a random selection of 50 patients was subtracted for aesthetic outcome evaluation. This group enclosed 25 patients from the AFT group and 25 from the IBR group.

#### Panels

A total of 30 evaluators completed the scoring, consisting of nine males and 21 females. Average age for all evaluators was 48.8 ± 11.2 years. All known characteristics of evaluators, divided per group, are shown in Table 2.

**Table 2.** Age and sex of evaluators.

Group	Male	Female	Age (mean ± SD), yr
A. Plastic surgeons	6	4	44.0 ± 8.7
B. Breast cancer patients	0	10	50.9 ± 13.1
C. Laymen	3	7	51.4 ± 11.0
Total	9	32	48.8 ± 11.2

### Descriptive Data

Mean age for all included subjects was 48.3 years (SD 9.8); mean BMI was 23.8 (SD 2.6). Furthermore, 15.0 (30.0%) patients received a bilateral reconstruction. Mamilla reconstruction was performed in 32 patients (64.0%) and a contralateral reconstruction was performed in 11.0 (22.0%) patients. The AFT group underwent more surgeries than the IBR group (4.76 ± 1.05 vs 1.96 ± 0.45). Patient characteristics per treatment group are presented in Table 3.

**Table 3.** Patient characteristics.

Characteristic	AFT (*n* = 25)	IBR (*n* = 25)	*p*-Value
Age (mean ± SD), yr	49.3 ± 9.6	47.2 ± 10.1	0.466
BMI (mean ± SD), kg/m^2^	23.9 ± 2.8	23.7 ± 2.5	0.742
*Laterality*^*1*^			0.208
Bilateral	6.0 (24.0%)	10.0 (40.0%)	
*Mamilla*^*2*^			0.070
Yes	13.0 (52.0%)	19.0 (76.0%)	
*Contralateral surgery*			–
Yes	6.0 (24.0%)	5.0 (20.0%)	
Surgeries (mean ± SD)	4.8 ± 1.1	2.0 ± 0.5	*< 0.001*

^1^Laterality of AFT reconstruction

^2^Presence of mamilla

### Aesthetic Outcome Scores

All mean VAS scores per evaluating group are presented in Table 4. The laymen group was the only panel with a statistical difference in mean scores between the treatment groups, with the AFT group scoring lower than the IBR group (− 1.04, *p* ≤ 0.001). No significant difference in mean scores was found for the other two panels, or when taking all scores into account. The plastic surgeons gave highest scores and the only group with a higher mean score for the AFT group.

**Table 4.** Mean VAS scores presented per panel

Group	AFT (mean score ± SD)	IBR (mean score ± SD)	Crude difference	*p*-Value
Plastic surgeons	7.12 ± 0.79	6.84 ± 0.78	+ 0.28	0.22
Breast cancer patients	5.78 ± 0.97	6.30 ± 0.97	− 0.51	0.068
Laymen	5.27 ± 1.08	6.31 ± 1.11	− 1.04	*<0.001*
All evaluators	6.06 ± 0.84	6.49 ± 0.89	− 0.24	0.087

Italic value indicate significance of p value (*p*<0.05)

### Agreement within the Groups

The ICC for each group is presented in Table 5. The highest ICC was for the laymen with an ICC of 0.45; the lowest ICC was for the plastic surgeons (ICC 0.34). All indicating poor agreement [15].

**Table 5.** Agreement within groups

Panel	ICC	95% confidence interval
Plastic surgeons	0.34	0.25–0.46
Breast cancer patients	0.36	0.26–0.49
Laymen	0.45	0.35–0.58
All evaluators	0.29	0.23–0.34

### Factors Influencing Scoring

For plastic surgeons, we found a positive correlation (*R*^2^ = 0.222) between reported scores and number of surgeries (*p* = 0.015) and bilateral reconstructions (*p* = 0.008). Breast cancer patients gave higher scores (*R*^2^ = 0.405) when the mamilla was present (*p* < 0.001) and lower scores if the subject was older (*p* = 0.005). The laymen also gave higher scores (*R*^2^ = 0.563) if the mamilla was present (*p* < 0.001) or if it was a bilateral reconstruction (*p* = 0.039), lower scores were given if the subject was older (*p* = 0.006).

## Discussion

This study was conducted to compare aesthetic outcome scores of implant-based reconstructions (IBR) with autologous fat transfer (AFT) breast reconstructions.

Our results showed breast cancer patients and plastic surgeons did not report different aesthetic scores for AFT and IBR. In line with previous studies, laymen were the lowest scorers. Additionally, this was the only group reporting significantly lower scores for the AFT group. Former studies evaluating subjective aesthetic outcomes argue that scores given by laymen are unreliably low due to their lack of experience with consequences of breast cancer, or achievable results with breast reconstruction post-mastectomy [9, 16]. In contrast, plastic surgeons are said to give higher scores, which are dependable due to their experience with multiple breast reconstruction and their knowledge on how bad and good results can be [17, 18]. Scoring behavior for breast cancer patients seems to be in-between the former groups and although some authors claim only plastic surgeons should be involved in aesthetic outcome evaluations due to their experience, we believe that breast cancer patients and laymen correlate more to the social network of breast cancer patients and thus should especially be included in these aesthetic outcome studies [17, 18].

Furthermore, it is stated that agreement between plastic surgeons is higher compared with inexperienced assessors [17, 18]. Our results do not correspond with these findings, seen as agreement was actually lower in the plastic surgeon group. Overall, agreement within all groups was poor, suggesting that cosmesis is indeed very personal and plastic surgeons should discuss patients’ preferences before the start of breast reconstruction [19].

Aesthetic outcome is a keystone of breast reconstruction for both patients and plastic surgeons [20–23]. Yet there is no standardized method to assess aesthetic outcome after breast reconstruction. Different methods available for cosmetic assessment have been thoroughly compared in a systematic review by Potter et al. [24] Evaluating methods used by healthcare professionals can be broadly categorized as clinical, photographic, and geometric. The majority of studies use the photographic method and have a panel of observers review images with the most frequently assessed views being frontal and oblique. Scoring itself is possible by using a VAS ranging from poor to excellent using a predefined point scale. Thus, in this study a VAS score was used for 3D photographic evaluation by three different panels.

Since our population included a variety of patients, e.g., presence of mamilla, laterality of reconstruction and age, a regression analysis was performed to determine possible factors influencing scoring. Interestingly, we found higher scores were given when a bilateral reconstruction was performed. This is a result that can be expected given the fact that symmetry is a key issue for the personal experience of aesthetics [9, 25] and that symmetry is more difficult to achieve when a unilateral reconstruction is performed because of the presence of a healthy breast as reference. Scores were also influenced by patient’s age. This further supports claims that aesthetic judgment is subjective.

An important rationale for lower scores reported for AFT reconstructions could be due to clinical differences. Although no statistical baseline differences were found between IBR and AFT group, there were six more patients with a mamilla in the IBR group and four more patients with a bilateral reconstruction in the IBR group. As shown by regression analysis, these clinical characteristics were predictors for higher scores. Hence, these differences in patient characteristics could have led to higher reported scores for the IBR group, since this group comprised more patients with positive clinical predictors.

Although we hypothesize aesthetic judgement is personal, research has shown social media to have significant impact on perceptions of ideal body image and aesthetic consults are often prompted by unrealistic images shown in pornographic magazines [26–28]. Body distortion and an unrealistic view on breasts could also be awakened by social media, portraying perky breasts, often enhanced with implants, as ideal breasts [29–31]. Especially for laymen, this factor could have influenced breast reconstruction rating seen as AFT breasts are often more natural-looking with ptosis. It is also pivotal to take cultural differences into account. Studies have reported differences in body perceptions, aesthetic scoring, and social media influence between countries. While perky and large breasts might have been considered ideal for our Dutch population, this might not be applicable to other ethnicities [32–34].

As with the majority of studies, our study suffered from limitations. First, subjects included varied in characteristics such as age, laterality of reconstruction and presence of mamilla. Analyses showed these characteristics influence scoring and thus more identical subjects could have corrected for differences in agreement. Furthermore, more evaluators per group are necessary to evaluate effect sizes. However, in reality, this setting is difficult to achieve. Additionally, 3D images were used for evaluation. Even though the quality of these images is superior to 2D images, positioning is not ideal. Arms of subjects are raised, possibly affecting shape of the breasts. A superior way, yet difficult setup, would be to include 3D video imaging.

Lastly, an overall score was examined, not questioning specific patient characteristics such as mastectomy scar, inframammary fold, ptosis grade, symmetry or shape. Implementing these subscales could identify important factors influencing scoring and lead us to a better understanding on how different individuals observe and judge breast contours [9]. This might be quite informative for developing patient education materials. Furthermore, the scale utilized was only labeled for the two extremes (one and ten), all scores in between were left undefined. This gave the evaluators more freedom for interpretation. At the same time, this could have led to larger differences within the groups since some evaluators might inherently give lower or higher scores. This might in turn have influenced correlation within the panels. Future studies should be warranted for this and consider predefining all points on the scale. Strengths of our study include the use of three independent evaluating panels, 3D imaging and random versions of presentations. Moreover, a large number of subjects from two treatment groups (25 per group) were compared.

Our results show aesthetic agreement is low within all evaluating groups and therefore a very subjective measurement that is difficult to measure. Seen as laymen gave the lowest scores and that this is probably due to their lack of experience with breast reconstructions, it could be that thorough patient counseling discussing achievable reconstruction results could alter satisfaction with outcome. These claims are supported by Ho et al. stating that patients’ satisfaction with breasts and overall outcome is dependent on preoperative information and interaction with plastic surgeon [35].

## Conclusion

This was the first study evaluating cosmetic results of AFT vs. IBR, using three evaluating panels. Results showed that laymen gave higher scores for the IBR group. No differences were found between the two techniques for the other evaluating groups. Overall scoring was low, and agreement within the groups was low. From these findings, we conclude that breast reconstruction cosmesis is perceived highly variable between individuals, and therefore, we encourage thorough patient counseling before starting reconstruction treatment.

## Protocol and Registration

This study has been registered at ClinicalTrials.gov (NCT02339779) and a published version of the protocol for the BREAST trial is available online [13].

## Disclaimer

The funder did not have any authority over any of the study-related activities, consisting of data collection, data management, data analysis, interpretation of results, writing the report, or submission for publication.

## Data Availability

The funder did not have any authority over any of the study-related activities, consisting of data collection, data management, data analysis, interpretation of results, writing the report, or submission for publication.

## Appendix A. STROBE Statement—Checklist of Items that Should be Included in Reports of Observational Studies

**Table:**

	Item no.	Recommendation	Page no.
Title and abstract	1	(a) Indicate the study’s design with a commonly used term in the title or the abstract	1, 2
		(b) Provide in the abstract an informative and balanced summary of what was done and what was found	2
*Introduction*			
Background/rationale	2	Explain the scientific background and rationale for the investigation being reported	3
Objectives	3	State specific objectives, including any prespecified hypotheses	3
*Methods*			
Study design	4	Present key elements of study design early in the paper	4
Setting	5	Describe the setting, locations, and relevant dates, including periods of recruitment, exposure, follow-up, and data collection	4
Participants	6	(a) *Cohort study*—Give the eligibility criteria, and the sources and methods of selection of participants. Describe methods of follow-up	4–5
		*Case-control study*—Give the eligibility criteria, and the sources and methods of case ascertainment and control selection. Give the rationale for the choice of cases and controls	
		*Cross-sectional study*—Give the eligibility criteria, and the sources and methods of selection of participants	
		(b) *Cohort study*—For matched studies, give matching criteria and number of exposed and unexposed	
		*Case-control study*—For matched studies, give matching criteria and the number of controls per case	
Variables	7	Clearly define all outcomes, exposures, predictors, potential confounders, and effect modifiers. Give diagnostic criteria, if applicable	5, 6
Data sources/measurement	8*	For each variable of interest, give sources of data and details of methods of assessment (measurement). Describe comparability of assessment methods if there is more than one group	5
Bias	9	Describe any efforts to address potential sources of bias	6
Study size	10	Explain how the study size was arrived at	6
Quantitative variables	11	Explain how quantitative variables were handled in the analyses. If applicable, describe which groupings were chosen and why	6
Statistical methods	12	(a) Describe all statistical methods, including those used to control for confounding	6
		(b) Describe any methods used to examine subgroups and interactions	6
		(c) Explain how missing data were addressed	N/A
		(d) Cohort study—If applicable, explain how loss to follow-up was addressed	
		Case-control study—If applicable, explain how matching of cases and controls was addressed	
		Cross-sectional study—If applicable, describe analytical methods taking account of sampling strategy	N/A
		(e) Describe any sensitivity analyses	N/A
Results			
Participants	13*	(a) Report numbers of individuals at each stage of study—e.g., numbers potentially eligible, examined for eligibility, confirmed eligible, included in the study, completing follow-up, and analyzed	7
		(b) Give reasons for non-participation at each stage	N/A
		(c) Consider use of a flow diagram	N/A
Descriptive data	14*	(a) Give characteristics of study participants (e.g., demographic, clinical, social) and information on exposures and potential confounders	7, Tables 1, 2
		(b) Indicate number of participants with missing data for each variable of interest	-
		(c) Cohort study—Summarize follow-up time (e.g., average and total amount)	7
Outcome data	15*	Cohort study—Report numbers of outcome events or summary measures over time	7,8, Tables 3, 4
		*Case-control study—*Report numbers in each exposure category, or summary measures of exposure	*–*
		*Cross-sectional study—*Report numbers of outcome events or summary measures	*–*
Main results	16	(a) Give unadjusted estimates and, if applicable, confounder-adjusted estimates and their precision (e.g., 95% confidence interval). Make clear which confounders were adjusted for and why they were included	7, 8, Tables 3, 4
		(b) Report category boundaries when continuous variables were categorized	–
		(c) If relevant, consider translating estimates of relative risk into absolute risk for a meaningful time period	–
Other analyses	17	Report other analyses done—e.g., analyses of subgroups and interactions, and sensitivity analyses	8
Discussion			
Key results	18	Summarize key results with reference to study objectives	8
Limitations	19	Discuss limitations of the study, taking into account sources of potential bias or imprecision. Discuss both direction and magnitude of any potential bias	10
Interpretation	20	Give a cautious overall interpretation of results considering objectives, limitations, multiplicity of analyses, results from similar studies, and other relevant evidence	10
Generalizability	21	Discuss the generalizability (external validity) of the study results	10
Other information			
Funding	22	Give the source of funding and the role of the funders for the present study and, if applicable, for the original study on which the present article is based	11

An explanation and elaboration article discusses each checklist item and gives methodological background and published examples of transparent reporting. The STROBE checklist is best used in conjunction with this article (freely available on the Web sites of PLoS Medicine at http://www.plosmedicine.org/, Annals of Internal Medicine at http://www.annals.org/, and Epidemiology at http://www.epidem.com/). Information on the STROBE Initiative is available at http:// www.strobe-statement.org

*Give information separately for cases and controls in case-control studies and, if applicable, for exposed and unexposed groups in cohort and cross-sectional studies
